# Esmarch’s Bandage in Anæsthesia

**Published:** 1886-12

**Authors:** 


### Esmarch's Bandage in Anæsthesia.

We have already noted that Dr. M. J. Roberts has found that the use of a tight rubber band about a part will greatly prolong the anæsthetic effect of cocaine; and now we read in the *Bull.*

*Gen. de Therap.* that M. Chandelux recommends the previous application of Esmarch's bandage when it is desired to produce local anæsthesia by means of ether spray. He was lead to make use of this precedure by the observations of M. Horand in 1867, who found that the difficulty of anæsthetizing a part bore a direct relation to its vascularity. The author claims the following advantages for his method:

1. Anæsthesia is produced in from twenty to forty seconds, while by the ordinary method two minutes or more are required.
2. After the spray is discontinued the anaesthesia remains for about three minutes, since the parts are not warmed by the blood-current.
3. The operation—for example, the removal of an in-growing nail—is greatly facilitated by the absence of hemorrhage.—*Medical and Surgical Reporter.*

